# The transporters SLC35A1 and SLC30A1 play opposite roles in cell survival upon VSV virus infection

**DOI:** 10.1038/s41598-019-46952-9

**Published:** 2019-07-18

**Authors:** Anna Moskovskich, Ulrich Goldmann, Felix Kartnig, Sabrina Lindinger, Justyna Konecka, Giuseppe Fiume, Enrico Girardi, Giulio Superti-Furga

**Affiliations:** 1 CeMM Research Center for Molecular Medicine of the Austrian Academy of Sciences, 1090 Vienna, Austria; 2 Center for Physiology and Pharmacology, Medical University of Vienna, 1090 Vienna, Austria

**Keywords:** Apoptosis, Mutagenesis

## Abstract

Host factor requirements for different classes of viruses have not been fully unraveled. Replication of the viral genome and synthesis of viral proteins within the human host cell are associated with an increased demand for nutrients and specific metabolites. With more than 400 acknowledged members to date in humans, solute carriers (SLCs) represent the largest family of transmembrane proteins dedicated to the transport of ions and small molecules such as amino acids, sugars and nucleotides. Consistent with their impact on cellular metabolism, several SLCs have been implicated as host factors affecting the viral life cycle and the cellular response to infection. In this study, we aimed at characterizing the role of host SLCs in cell survival upon viral infection by performing unbiased genetic screens using a focused CRISPR knockout library. Genetic screens with the cytolytic vesicular stomatitis virus (VSV) showed that the loss of two SLCs genes, encoding the sialic acid transporter SLC35A1/CST and the zinc transporter SLC30A1/ZnT1, affected cell survival upon infection. Further characterization of these genes suggests a role for both of these transporters in the apoptotic response induced by VSV, offering new insights into the cellular response to oncolytic virus infections.

## Introduction

Solute carriers (SLCs) are the largest family of transporters in the human genome and the second largest family of transmembrane proteins after G-protein coupled receptors (GPCRs). As such, they represent the major “gatekeepers” of energy supply and metabolism in a cell, controlling the uptake and release of a growing list of small molecules and metabolites^1^. Despite a large number of links between SLCs and human diseases, this family remains significantly understudied, as ~30% of the roughly 400 family members have a completely unknown function(s) or substrate specificity^1,2^.

Viruses represent a major class of pathogens, acting as potent and pleiotropic modulators of cellular metabolism and function. This can, in turn, be harnessed to explore the biology of cellular and immunological processes. Given their complete dependence on host metabolism for replication, viruses have been shown to dramatically alter and exploit cellular pathways for their purpose^3^. Accordingly, various transporters have been found to be modulated during virus infection^4^, required as viral receptors^5,6^ or important for viral entry into the cell^7,8^. Finally, solute carriers can play important roles in determining the outcome of anti-viral immune responses^9,10^.

In this study, we used an unbiased genetic screen to identify transporters affecting infectivity and cellular survival upon treatment with influenza virus (IAV) or vesicular stomatitis virus (VSV). In particular, we focused on VSV, which has been extensively used as a model system when studying virus replication and the viral life cycle^11^. As a cytolytic virus, VSV rapidly and effectively induces cell death via intrinsic and extrinsic apoptotic pathways^12^. Due to its non-pathogenic nature in humans and its ability to rapidly induce apoptosis in infected cancer cells, VSV has shown promise in cancer immunotherapy^13^ and oncolytic virotherapies^14^, as well as in the development of vaccines, e.g. against Ebola virus^15^. Hence, there is significant interest in determining the host factors affecting cell survival upon VSV infection. Here we describe two transporters, the sialic acid transporter SLC35A1 and the zinc transporter SLC30A1 (ZnT1), as factors promoting either resistance or sensitivity upon VSV infection.

## Results

### An SLC-focused CRISPR knockout genetic screen identifies SLC35A1 and SLC35A2 as factors required for IAV infection

**Figure 1:**
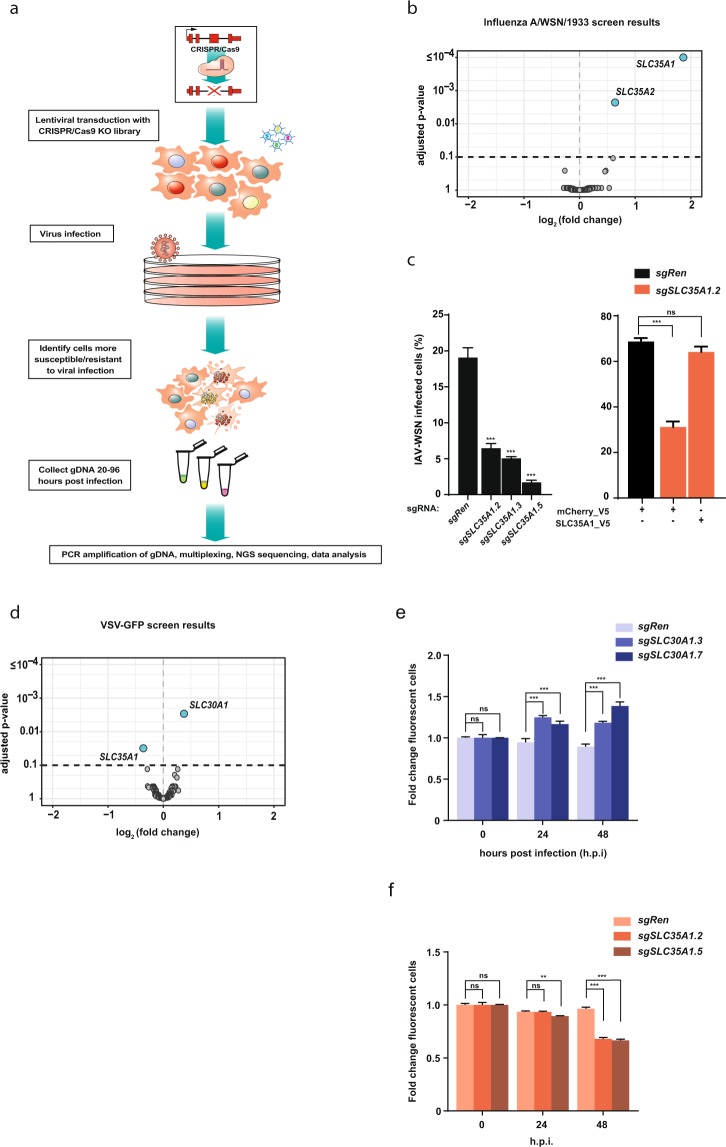
An SLC-focused genetic screen identifies known and novel host factors for IAV and VSV. (**a**) Schematic overview of the screen workflow. (**b**) Volcano plot of the genetic screen results in A549 cells infected with IAV-WSN/1933 at 96 h.p.i. (**c**) Quantification of flow cytometry-based viral replication assay for selected hits. A549 cells carrying sgRNAs targeting *SLC35A1* or *Renilla luciferase* control as well as cells expressing SLC35A1 cDNA were infected with IAV-WSN/1933 at MOI 0.01 and 24 h.p.i. the number of the infected cells was assessed after staining with anti-Influenza Nuclear protein antibody coupled to AF488. (**d**) Volcano plot of the genetic screens results in A549 cells infected with VSV-GFP at 48 h.p.i. (**e**,**f**) Multicolor competition assay (MCA) of *SCL30A1* (**e**) or *SLC35A1* (**f**) knockout cells and *Renilla* controls. Cells were mixed at 1:1 ratio, infected with VSV-rWT at MOI 5 and cultured until the indicated time points. The percentage of mCherry + (*Renilla* control) and eGFP^+^ (gene of interest) cells in the live population (FSC/SSC) were assessed using flow cytometry. Statistical significance was calculated using one-way ANOVA (**c**) and two-way ANOVA (**e**,**f**) with Dunnett’s test. Unless otherwise indicated, adjusted P-values in relation to the sgRen control are shown. Data are represented as mean ± SD of one representative experiment out of at least two independent replicates. **p ≤ 0.01; ***p ≤ 0.001; ns: not significant.

In order to identify solute carriers affecting viral infection and cell survival, we performed unbiased genetic screens using an SLC-focused CRISPR knockout lentiviral library targeting 388 SLC genes with multiple sgRNAs per gene^16^ (Fig. 1a). To test the ability of this library to identify factors affecting viral infection, we screened for transporters affecting survival of the lung adenocarcinoma cell line A549 upon influenza A virus (IAV, strain A/WSN/1933) infection. A549 cells were transduced with the SLC knockout library and subsequently infected with IAV at the MOI of 0.5. Samples were collected 96 hours post-infection (h.p.i.) after which the library compositions of the treated and untreated samples were compared through the sequencing of sgRNA inserts. We observed good representation of the library in all samples (Suppl. Fig. 1a), and good correlations between replicates (Suppl. Fig. 1b). We identified two SLC genes, *SLC35A1* and *SLC35A2*, whose sgRNAs were significantly enriched in the surviving cell population (Fig. 1b, Suppl. Table 1). Both genes are members of the large SLC35 family of nucleotide-sugar transporters and the two corresponding proteins localize to the Golgi apparatus^17^, with SLC35A1/CST acting as a CMP-sialic acid/CMP antiporter and SLC35A2 as a UDP-galactose/UMP antiporter. IAV exploits sialic acid, a hexose residue present on several oligosaccharides, as its main cell entry receptor^18^. Lack of SLC35A2 (which results in the loss of the galactose residue often present upstream of the terminal sialic acid) or SLC35A1 has been previously shown to cause reduced or abolished levels of sialylation on the cell surface, resulting in a severe impairment of IAV docking and entry^19,20^. Consistent with the screening results, CRISPR/Cas9-based knockout of the *SLC35A1* or *SLC35A2* genes in A549 cells increased resistance to IAV infection (Fig. 1c, Suppl. Fig. 1c,d). The effect due to the inactivation of *SLC35A1* could be reversed by ectopic expression of SLC35A1 (Fig. 1c, Suppl. Fig. 1e), confirming the dependence of the observed phenotype on this specific gene. Altogether, this set of experiments validated the use of our SLC-focused CRISPR knockout library as well as our experimental approach to investigate the role of SLC genes in the viral life cycle. Moreover, it confirmed the non-redundant role of members A1 and A2 of the SLC35 family, among all other SLCs represented in the library, in determining the ability of influenza A virus to infect human cells.

### Genetic screening identifies SLC35A1 and SLC30A1 as modulators of cell survival upon VSV infection

A549 cells showed substantial cell death starting at 24 hours post VSV infection (above 50% cell death at MOI 10) (Suppl. Fig. 1f). For the genetic screen, A549 cells were infected with the SLC library and subsequently infected with VSV virus at an MOI of 10 (Fig. 1a). Samples were collected pre infection and at 48 h.p.i. As before, we observed good representation of the library in all samples (Suppl. Fig. 1g), and good correlations between replicates (Suppl. Fig. 1h). Comparison of the sgRNA abundance between the virus-treated and control samples at 48 h.p.i. showed a significant differential enrichment for two transporters: *SLC30A1* (ZnT1, log_2_ fold change: 0.373, adjusted *p*-value: 7 × 10^−6^) and *SLC35A1* (log_2_ fold change: −0.362, adjusted *p*-value: 1.5 × 10^−4^) (Fig. 1d). Interestingly, loss of SLC35A1, which conferred resistance in the IAV screen, resulted in increased cell susceptibility to the VSV infection. Moreover, we identified SLC30A1, which is the only member of the SLC30 family reported to export zinc at the plasma membrane^21^, as a permissive factor. In order to validate these results, we used a multicolor competition assay (MCA) approach in A549 cells carrying sgRNAs targeting *SLC30A1*, *SLC35A1* or expressing a sgRNA targeting *Renilla luciferase* as negative control. We observed a significant enrichment of cells carrying SLC30A1 sgRNAs compared to control cells at both 24 and 48 hours after infection, validating the outcome of the screen and suggesting that SLC30A1 is, in fact, exerting a negative effect on cell survival upon VSV infection (Fig. 1e, Suppl. Fig. 1i). Conversely, inactivation of the *SLC35A1* gene resulted in strongly increased cell death compared to the control at 48 h.p.i., suggesting that this transporter supports cell survival after VSV infection, thereby validating the primary screen (Fig. 1f, Suppl. Fig. 1j).

### Loss of the Zn-exporter SLC30A1 inhibits caspase activation upon VSV virus infection

**Figure 2:**
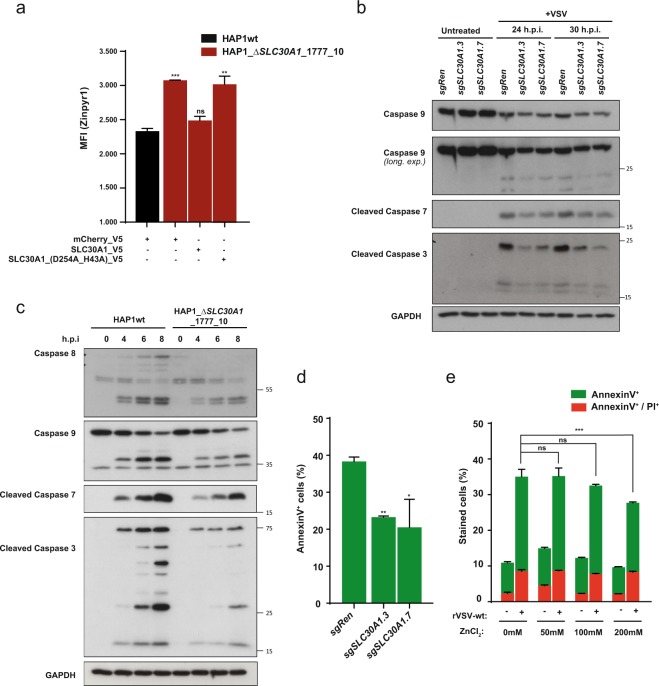
SLC30A1 inhibits apoptotic caspase activation via regulation of intracellular Zn^2+^ levels. (**a**) Intracellular Zn^2+^ levels determined using Zinpyr1 fluorescent dye. (**b**,**c**) Activation of pro-apoptotic caspases in VSV infected A549 (**b**) and HAP1 (**c**) *SLC30A1* knockout cells. Cropped images are shown for conciseness. Full-length blots are presented in Supplementary Fig. 5. (**d**) Percentage of apoptotic cells in A549 cells expressing sgRNAs targeting *SLC30A1* or *Renilla luciferase* after infection with VSV (MOI 2) for 24 hours, as measured with AnnexinV-FITC. (**e**) Quantification of AnnexinV- and PI-stained A549 wild type cells pre-treated with indicated concentrations of ZnCl_2_ and infected with VSV-rWT (MOI 2) for 24 hours. Statistical significance was assessed using one-way ANOVA (**a**,**d**) and two-way ANOVA (**e**) with Dunnett’s test. Unless otherwise indicated, adjusted P-values in relation to the sgRen control or Hap1wt are shown. Data are represented as mean ± SD of one representative experiment out of at least two independent replicates. *p ≤ 0.05; **p ≤ 0.01; ***p ≤ 0.001; ns: not significant.

SLC30A1 has been reported to be responsible for the export of zinc from the cytoplasm^21,22^. To confirm its role in the homeostasis of zinc levels in our cellular system, we measured intracellular levels of zinc using the Zinpyr1 dye^23^. A549 cells lacking SLC30A1 (Suppl. Fig. 2a) or a single-cell-derived HAP1 *SLC30A1* knockout clone (Δ*SLC30A1*_1777_10) showed increased levels of zinc compared to control cell lines (Fig. 2a, Suppl. Fig. 2b). This effect was reversed by ectopic expression of SLC30A1 (Fig. 2a, Suppl. Fig. 2c,d) in the HAP1 SLC30A1-deficient cells. Moreover, a transport-deficient mutant of SLC30A1 (D254A_H43A)^24^, failed to decrease intracellular zinc levels (Fig. 2a) despite localization patterns similar to the wild type protein (Suppl. Fig. 2c,d). These results therefore support the role of SLC30A1 as a zinc exporter. Zn^2+^ is directly implicated in regulating the activity of several pro-apoptotic caspases such as caspase-3, -6, -7, -8 and -9^25–27^. Since VSV is a cytolytic virus, able to kill infected cells via induction of apoptosis through both intrinsic and extrinsic pathways^14^, we speculated that elevated intracellular zinc levels might interfere with the activation of pro-apoptotic signalling through inhibition of caspases. To experimentally verify this hypothesis, we monitored the cleavage of different apoptotic caspases in VSV-infected A549 and HAP1 cells (Fig. 2b,c). Indeed, A549 cells lacking the SLC30A1 transporter had diminished levels of all expressed and cleaved caspases tested (caspase-3, -7, -9) (Fig. 2b). This effect was even more pronounced in the HAP1 *SLC30A1*-deficient clone (Fig. 2c). Consistent with diminished caspase activation, we observed significantly reduced numbers of Annexin V-positive *SLC30A1* knockout cells compared to wild type A549 cells (Fig. 2d). Reduced numbers of Annexin V-positive cells were also observed in the HAP1 SLC30A1-deficient clone (Suppl. Fig. 2e) and this effect could be reversed by ectopic expression of wild type SLC30A1, but not by the transport-deficient mutant (Suppl. Fig. 2e–g). In line with this data, we observed a reduction of Annexin V- positive cells upon VSV infection in wild type cells supplemented with Zn^2+^ (ZnCl_2_ concentrations above 100 mM) (Fig. 2e). Altogether, these data show that loss of *SLC30A1* in A549 and HAP1 cells results in increased intracellular levels of Zn^2+^ and that cells lacking this transporter show reduced caspase activation and apoptotic cell death upon VSV infection.

### SLC30A1-deficient cells show reduced infectivity in multicycle infections

**Figure 3:**
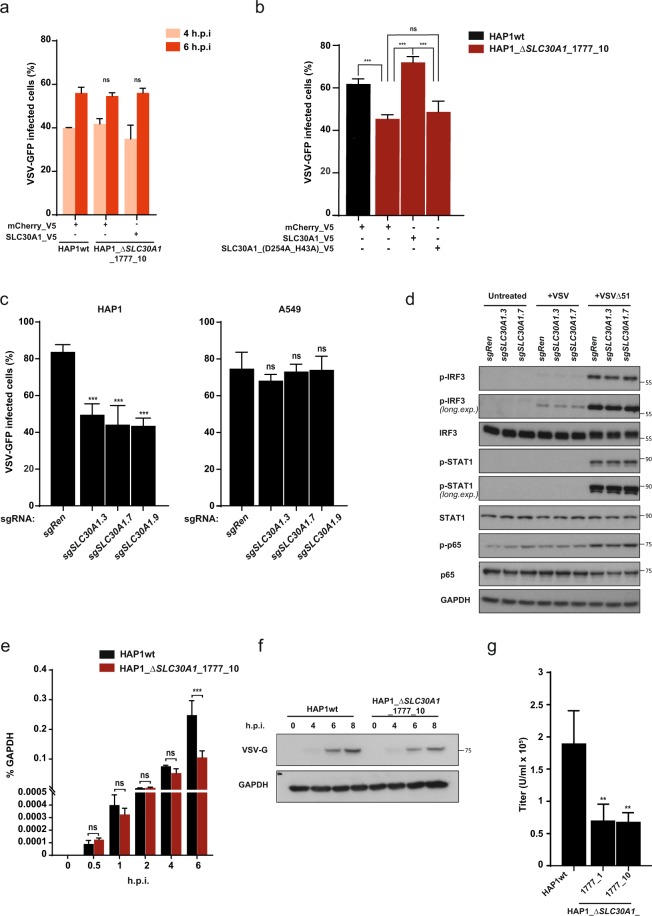
SLC30A1 loss affects VSV infection through modulation of Zn levels. (**a**–**c**) VSV-GFP virus replication in *SLC30A1* knockout HAP1 cells (**a**,**c**) and HAP1 cells overexpressing wild type SLC30A1 and SLC30A1(D254A_H43A) cDNAs (**b**) and A549 cells (**c**). Cells were infected with MOI 1 (**a**), MOI 0.001 (**b**) and MOI 0.002 (**c**). The number of GFP+ cells was quantified using flow cytometry at 14 h.p.i. (**b**,**c**) or indicated time points (**a**). (**d**) Immunoblot analysis of type I IFN/STAT1 signalling pathway activation in the VSV-infected (MOI 2) HAP1 cells carrying sgRNAs against *SLC30A1* and *Renilla luciferase* control. Cropped images are shown for conciseness. Full-length blots are presented in Supplementary Fig. 5. (**e**) Quantitative RT-PCR of total viral RNA in the HAP1 wild type cells and a *SLC30A1* single cell knockout clone at different points after VSV infection (MOI 2). (**f**) VSV-G protein levels in the HAP1 wild type cells and *SLC30A1* single cell knockout clone at different points after VSV infection (MOI 2). Cropped images are shown for conciseness. Full-length blots are presented in Supplementary Fig. 5. (**g**) Quantification (flow cytometry) of virus titers produced by HAP1wt and two *SLC30A1* single cell knockout clones infected with VSV-GFP for 8 hours at MOI of 2. Statistical significance was assessed using two-way ANOVA (**a**,**e**) and one-way ANOVA (**b**,**c**,**g**) with Dunnett’s (**a**,**c**,**g**), Tukey’s (**b**) or Sidak’s correction (**e**) tests. Unless otherwise indicated, adjusted P-values in relation to the sgRen or HAP1wt control are shown. Data are represented as mean ± SD of one representative experiment out of at least two independent replicates. **p ≤ 0.01; ***p ≤ 0.001; ns: not significant.

We next investigated which stage of the VSV life cycle is affected by the loss of *SLC30A1*. Infection of HAP1 cells at MOI > 1 did not result in altered percentages of infected cells in *SLC30A1* HAP1 knockout cells at early time points (Fig. 3a), indicating that loss of this transporter did not impair viral entry. However, upon low («1) MOI VSV infection at later time points (which resulted in multicycle infections) we observed a strong reduction in the number of infected cells (Fig. 3b). The reduction in infectivity associated with *SLC30A1* loss in HAP1 cells could be reverted by overexpression of the transport-competent form of SLC30A1. In contrast, the D254A_H43A transport mutant failed to rescue the viral phenotype, suggesting that the transport function was required (Fig. 3b). The reduction in infectivity was further confirmed in independently generated populations of HAP1 and HEK293T cells transduced with different sgRNAs targeting *SLC30A1*, although A549 did not show similar results (Fig. 3c, Suppl. Fig. 3a,b). We did not observe any reduction in infectivity upon treatment with the pan-caspase inhibitor z-VAD-FMK, which reduces cleavage of caspase-3 to levels comparable to the *SLC30A1* knockout (Suppl. Fig. 3c,d). This is in line with previous observations suggesting that, while inhibition of caspases may temporarily attenuate VSV-induced apoptosis, it does not actually affect virus replication^28,29^. Given the well-described role of Zn^2+^ ions and other zinc transporters in the immune response against different pathogens and inflammatory stimuli^30^, we set out to test whether *SLC30A1* loss may affect the antiviral immune response against VSV infection. Wild type VSV does not generally elicit a strong immune response (Fig. 3d). In contrast, VSV harbouring mutations to a matrix (M) protein creates an attenuated form that fails to shut down host cell innate immune signalling efficiently and therefore elicits a stronger immune response. One of these mutants, VSV-∆M51-GFP^31^, caused a readily measurable response upon infection of HAP1 wild type cells, as shown by phosphorylation of IRF3 and STAT1 transcription factors (Fig. 3d). Similar to wild type VSV, loss of SLC30A1 resulted in reduced infection upon treatment with VSV-∆M51-GFP (Suppl. Fig. 3e). However, cells deficient for *SLC30A1* did not show notable differences in the activation of IFNα/β and NF-κB pathways, two of the major antiviral immune response pathways, when compared to wild type cells (Fig. 3d). Moreover, we did not detect differences in either accumulation of viral RNA (0.5–4 h) or viral protein (i.e. VSV-G, 6 h) at early time points (Fig. 3e,f). Interestingly, when measuring the titers of virus produced by the *SLC30A1* knockout cells compared to HAP1 wild type we did measure a significant reduction in the number of viral particles released from the *SLC30A1*-deficient cells (Fig. 3g). This is consistent with the reduced amount of viral RNA in the *SLC30A1*-deficient cells at the later stages of the infection (>4 hours), when reinfection by the produced viral particles starts to occur (Fig. 3e). Together, these observations suggest that SLC30A1-deficiency affects VSV at late stages of the virus life cycle. Overall, we did not observe effects on cell infectivity or immune response due to *SLC30A1*-loss under single-cycle conditions (MOI > 1). Interestingly, *SLC30A1*-deficient cells produced significantly reduced titers of virus particles after infection, likely due to a defect at the late stage of the virus cycle, which could become relevant in a multicycle study.

### Loss of SLC35A1 results in increased cell death and apoptosis upon VSV infection

**Figure 4:**
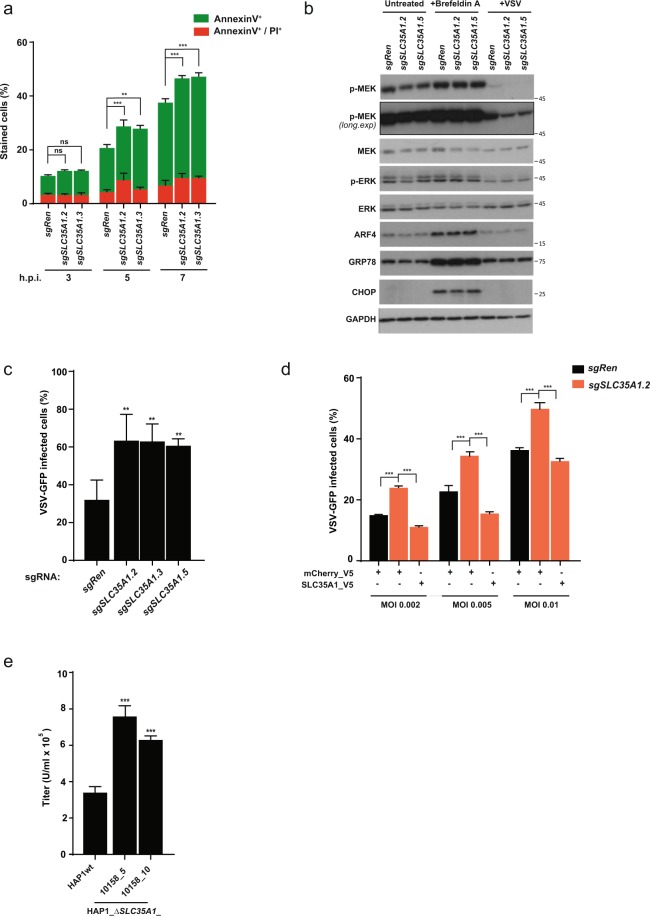
SLC35A1 loss promotes cell death and apoptosis upon VSV infection. (**a**) Percentage of early apoptotic cells (AnnexinV^+^) and cells that lost membrane integrity due to a late apoptotic/necrotic state (AnnexinV^+^/PI^+^) in HAP1 cell lines expressing sgRNAs against *SLC35A1* and *Renilla luciferase* upon infection with VSV at MOI 2. (**b**) Immunoblot analysis of Golgi stress signalling pathway activation in the VSV-infected (MOI 2) and Brefeldin A-stimulated (3μg/ml) A549 cells carrying sgRNAs against *SLC35A1* and *Renilla luciferase*. Cropped images are shown for conciseness. Full-length blots are presented in Supplementary Fig. 5. (**c**,**d**) VSV-GFP virus replication in *SLC35A1* knockout HAP1 cells (**c**) and HAP1 cells overexpressing SLC35A1 cDNA (**d**) infected with MOI ranging from 0.001 to 0.01 for 14 hours. (**e**) Quantification (flow cytometry) of virus titers produced by HAP1wt and two *SLC35A*1 single cell knockout clones infected with MOI of 2 of VSV-GFP for 8 hours. Statistical significance was assessed using one-way ANOVA with Tukey’s (**a**,**d**) and Dunnett’s (**c**,**e**) tests. Unless otherwise indicated, adjusted P-values in relation to sgRen control or HAP1wt are shown. Data are represented as mean ± SD of one representative experiment out of at least two independent replicates. **p ≤ 0.01; ***p ≤ 0.001; ns: not significant.

The sialic acid-CMP transporter SLC35A1 has previously been shown to be involved in the cellular response to apoptotic stimuli. In particular, *SLC35A1* loss has been reported to evoke an elevated Golgi stress response^32^ resulting in increased rates of pro-apoptotic signalling in the cells^30,33,34^. We therefore hypothesised that loss of SLC35A1 may result in sensitisation to cell death upon VSV infection. Indeed, we observed that A549 and HAP1 cells lacking SLC35A1 (Suppl. Fig. 4a) show elevated rates of cell death (Fig. 1f, Suppl. Fig. 1j) and apoptosis during VSV infection, as indicated by the increased number of Annexin V-positive cells (Fig. 4a,) and increased caspase cleavage (Suppl. Fig. 4b). These effects could be reversed by ectopic overexpression of *SLC35A1* (Suppl. Fig. 4b,c), suggesting a dependency on SLC function. Similar patterns in cell death were observed upon stimulation with Brefeldin A, a compound previously reported to induce a Golgi stress response^35^ (Suppl. Fig. 4d). However, cells lacking *SLC35A1* did not exhibit an increase in MAPK pathway activity or ARF4 protein levels, both of which have been reported to indicate a response to Golgi stress (Fig. 4b)^35^, suggesting the involvement of an alternative pathway(s). Consistent with this, we found that *SLC35A1* knockout cells were also more sensitive to the non-Golgi stress-related, cytotoxic compound camptothecin (Suppl. Fig. 4d). Interestingly, *SLC35A1* knockout cells showed increased infection rates in multicycle infections in several different cell lines (Fig. 4c, Suppl. Fig. 4e). This effect could also be reverted by re-expression of *SLC35A1* (Fig. 4d). Finally, we detected significantly increased virus titers in *SLC35A1* knockout cells compared to wild type cells following a single infection cycle, as measured by FACS (Fig. 4e), suggesting increased rates of viral replication. Altogether, these data suggest the decreased viability of cells lacking *SLC35A1* after viral infection arises from a combination of a more pronounced apoptotic response and an increased rate of VSV replication.

## Discussion

Functional genomic and genetic screens, based on siRNA, insertional mutagenesis and, more recently, CRISPR/Cas9, have been extremely valuable in identifying host factors affecting viral infectivity and replication, in particular regarding entry factors^19,20,36–38^. However, the role of transporters as host genes affecting both the viral life cycle and host cell survival upon infection remains relatively understudied. Here we employed SLC-focused CRISPR knockout screens to identify two solute carriers, the CMP-sialic acid/CMP transporter *SLC35A1*/CST and the zinc transporter *SLC30A1*/ZnT1 as critical host factors affecting the survival of human cell lines towards infection with the oncolytic VSV virus in opposite ways (Fig. 1). We confirmed that SLC30A1 is a zinc exporter localized at the plasma membrane and that genetic inactivation resulted in increased intracellular zinc levels and increased resistance to VSV-induced cellular killing (Figs 1–2). Mechanistically, loss of *SLC30A1* did not appear to affect the anti-viral immune response or viral entry (Fig. 3). We instead observed a reduced infection of *SLC30A1*-deficient cells compared to wild type cells in multicycle infections. This phenotype was also linked to reduced virus titers, potentially suggesting a defect in the later stages of the viral cycle (Fig. 3). More importantly, we observed a marked reduction of caspase activation and reduced apoptotic cell death upon *SLC30A1* inactivation (Fig. 2). Given the known role of Zn^2+^ ions as pan-caspase inhibitors^25–27^, we hypothesise that loss of SLC30A1, and the subsequent increase in intracellular Zn^2+^ levels, results in an apoptosis-resistant state and increased survival upon further perturbations, such as VSV infection.

The second transporter gene identified in our VSV screen was the Golgi-residing *SLC35A1*. This gene has previously been implicated in IAV infectivity due to its critical role in the addition of sialic acid, the major receptor of influenza virus, to proteins and lipids on the cell surface^39,40^. When we screened for solute carriers affecting IAV infectivity, we identified not only *SLC35A1* but also the related transporter *SLC35A2*, an UDP-galactose/UMP transporter, as hits (Fig. 1). These transporters have been previously shown to play an important role in determining the glycosylation pattern of secreted and plasma membrane proteins and are associated with congenital disorders related to glycosylation^41,42^. Moreover, both genes were previously described in genome-wide screens as host factors required for IAV infectivity^19,20^ and recapitulated in our screen. Interestingly, loss of *SLC35A1* led to increased cell death upon VSV infection (Figs 1 and 4). However, we did not observe activation of signalling pathways commonly associated with the induction of Golgi stress, a pro-apoptotic pathway previously shown to be linked to SLC35A1^32^. It was previously reported that sialic acid modifications play important roles for various pro-apoptotic signals^43–45^ as well as prevent apoptosis induced by galectins^44^. Further studies will be required to characterize which pathway(s) are involved in VSV- and SLC35A1-dependent cell death and viral phenotypes.

In conclusion, we used an SLC-focused genetic approach to identify transporter proteins affecting the survival of human cancer cell lines upon VSV infection. Both genes identified, *SLC30A1* and *SLC35A1*, represent novel host factors that affected the induction of apoptosis upon viral infection. These results offer new insights and may spark new ideas into possible strategies to pharmacologically interfere with viral infections, especially in the context of oncolytic viruses.

## Methods

### Cell lines and viruses

HEK293T and A549 cells were obtained from ATCC (Manassas, VA, USA), MDCK2 cells were kindly provided by G.Versteeg (MFPL, University of Vienna, Austria). HAP1 cells were obtained from Horizon Genomics. Cells were cultured in DMEM or IMDM medium supplemented with 10% (v/v) FBS and antibiotics (100 U/ml penicillin and 100 mg/ml streptomycin) (Gibco). Cell lines were routinely checked for mycoplasma using MycoAlert kit (Lonza). The virus strains used in this study were recombinant VSV-GFP^46^, VSV-rWT^47^, VSV-∆M51-GFP^31^ and IAV-WSN/1933 (H1N1). Viruses were grown on MDCK2 and Vero cells (both grown in DMEM supplemented with 10% FBS and antibiotics) and titrated using plaque assay. SLC35A1 and SLC30A1 single cell knockout HAP1 clones were obtained from Haplogen Genomics (SLC30A1 clones: 1777-1 and 1777-10, SLC35A1 knockout clones: 10158-5, 10158-10).

### CRISPR-knockout screening

The human SLC knockout CRISPR/Cas9 library used in this study has been described previously^16^. Viral particles were prepared by transient transfection of low passage, subconfluent HEK293T cells with the library plasmid pool together with packaging plasmids psPAX2 (Addgene #12260) and pMD2.G (Addgene #12259) using PolyFect (Qiagen). After 24 hours the media was changed to fresh RPMI media supplemented with 10% FCS and antibiotics. The viral supernatant was collected after 48 hours, filtered (0.45 μm) and stored at −80 °C until further use.

To determine the degree of cell survival upon VSV infection in A549 cells prior to the screen, cells were infected with VSV-GFP (MOI of 10) for the duration of 24 to 48 hours, fixed with 4% PFA in PBS, stained with 1% Crystal violet, solubilised with 70% Ethanol and absorbance was measured using spectrophotometer at 570 nm.

A549 cells were infected with the SLC knockout library at a multiplicity of infection of 0.2–0.3 and after selection for 5 days with puromycin (2 μg/ml) and 2 days of recovery and expansion, after which they were screened. For the screen, 8 × 10^6^ (equals to ~3000x coverage) of A549 cells were seeded in duplicates and infected with IAV-WSN and VSV-GFP at the MOI of 0.5 and 10 respectively. Cells were collected 96 hours post infection (for the IAV-WSN screen) and at 48 h hours post infection (for the VSV-GFP screen) together with time-matched uninfected controls. Additionally, time 0 samples were collected at the time of infection. Genomic DNA was extracted using the DNAeasy kit (Qiagen) and amplified with 2 rounds of PCR using primers derived from Shalem *et al*.^48^ and modified to allow dual indexing by addition of an additional barcode on the forward primers. The PCR-amplified samples were sequenced on a HiSeq2500 (Illumina) at the Biomedical Sequencing Facility (CeMM/Medical University of Vienna).

### Screen analysis

Sequencing reads were matched to the sgRNA library sequences and counted using an in-house script. Guide-level read counts were input to a gene-level DESeq2 model^49^. Custom normalization factors were provided, assuring equal median sgRNA read counts per sample. As the gene-level model included the effect of the guides, an identical number of guides (i.e. 6) per gene were required across all genes. Therefore, for 10 genes with only 5 guides in the library, a randomly selected guide was duplicated. For 3 genes with more than 6 guides in the library, as well as the negative control, 6 guides were randomly selected. The model was tested for differential abundance contrasting virus-infected samples versus the uninfected control samples of corresponding time points. Resulting p-values were corrected for multiple testing using the Benjamini-Hochberg procedure^50^ and genes with an adjusted p-value of less than 0.1 were considered significantly enriched or depleted, corresponding to an estimated false-discovery rate below 10%.

### Plasmids and knockout cell lines generation

For CRISPR-based knockout cell lines, sgRNAs were designed using CHOPCHOP^51^ and cloned into pLentiCRISPRv2 (Addgene, #52961), pLentiCRISPRv3^52^, pX459 (Addgene #48139), LGPIG (pLentiGuide-PuroR-IRES-GFP) or LGPIC (pLentiGuide-PuroR-IRES-mCherry)^53^. sg*Ren* targeting *Renilla luciferase* cDNA was used as negative control sgRNA (Suppl. Table 2)^53^. SLC35A1 (HsCD00415788) and SLC30A1 (HsCD00375357) cDNAs were obtained as gateway-compatible pENTR vectors from the Harvard PlasmID Repository. sgRNA-resistant cDNA versions as well as the SLC30A1 D254A_H43A double mutant^24^ were generated using NEB Q5 site-directed mutagenesis kit (Suppl. Table 2). cDNAs were transferred into the Gateway-compatible lentiviral expression vectors pLX304 (Addgene, #25890, CMV promoter driven expression) or pRRL (described previously^53^, EF1a promotor driven expression) using LR recombination (ThermoFisher Scientific). For the generation of HAP1 cells transiently expressing pRRL vectors carrying SLC30A1 wild type and D254A_H43A double mutant cDNA fused to mScarlet, cells were transfected using TurboFectin (Origene) 36 hours before measurement. For the generation of lentiviral knockout and overexpression cells HEK293T cells were transfected with psPAX2 (Addgene #12260) and pMD2.G (Addgene #12259) and expression vectors using Polyfect (Qiagen). 24 hours post transfection medium was replaced with fresh medium that was harvested 48 hours later, filtered (0.45 μm), supplemented with 5 μg/ml Polybrene (Hexadimethrine bromide, Sigma) and added to target cells. 48 hours after transduction the medium was supplemented with the respective selection antibiotics. Cells were selected for 5–7 days. Due to the downregulation of the expression of wild type cDNA of SLC30A1 over time, fresh overexpression cells were prepared every 2–3 weeks. Editing efficiency of sgRNAs was assessed by Sanger sequencing followed by analysis of sequencing results with TIDE web tool^54^. The following sgRNAs were used throughout the study:

*sgRen*:

**F:** CACCGGTATAATACACCGCGCTAC; **R**: AAACGTAGCGCGGTGTATTATACC

*sgSLC35A1.2*:

**F:** CACCGCTGGCGTCTACTTGTCAGA; **R**: AAACTCTGACAAGTAGACGCCAGC

*sgSLC35A1.3*:

**F:** CACCGCTGGAGTTACGCTTGTACA; **R**: AAACCTGTACAAGCGTAACTCCAGC

*sgSLC35A1.5*:

**F:** CACCGACATACAAGAAGAGTACCCA; **R**: AAACTGGGTACTCTTCTTGTATGTC

*sgSLC30A1.3*:

**F:** CACCGCGGCTCGATGAAGCGCTCGA; **R**: AAACTCGAGCGCTTCATCGAGCCGC

*sgSLC30A1.7*:

**F:** CACCGGCTGGACAACTTAACATGCG; **R**: AAACCGCATGTTAAGTTGTCCAGCC

*sgSLC30A1.9*:

**F:** CACCGGATCCGAGCCGAGGTAATGG; **R**: AAACCAGCGCTGTTCTCCGTGCTC

*sgSLC35A2.94*:

**F:** CACCGAGCACGGAGAACAGCGCTG; **R**: AAACCAGCGCTGTTCTCCGTGCTC

*sgSLC35A2.118*:

**F:** CACCGCTACGCCCGCACGTTGCCAG; **R**: AAACTGGCAACGTGCGGGCGTAGC

### Flow cytometry

For flow cytometric analyses of virus-infected cells, cells were seeded into 24-well plates and infected with IAV or VSV at the specified MOIs. Cells were collected at indicated time points and fixed with 4% PFA in PBS. IAV-WSN/1933 infected cells were permeabilized (PBS, 0.1% Triton X-100), stained with AlexaFluor-488-labelled (ThermoFisher Scientific) anti-influenza nucleoprotein antibody (ab20343, Abcam) for 1 hour at room temperature followed by two washing steps.

In order to determine the amount of dead or apoptotic cells after virus infection, cells were seeded into 12-well plates, infected at specified MOIs, and at the indicated time points. Depending on the experimental conditions, cells were stained with AnnexinV-FITC (eBioscience) or AnnexinV-AF647 (eBioscience), LIVE/DEAD™ Red or Green fixable dyes (Invitrogen) or Propidium Iodide (PI) for 15 min (AnnexinV and PI) or 30 min (LIVE/DEAD™) at room temperature, fixed with 4% PFA and subsequently analysed by flow cytometry.

To measure intracellular Zn^2+^ levels, cells were incubated for 30 min at 37 °C in serum-free IMDM supplemented with 2 mM EDTA and 50 μM Zinpyr 1 (Abcam, ab145349).

Flow cytometry-based multicolor competition assays (MCA) were performed as described previously^53^. Briefly, A549 cells expressing LGPIC-sgRen were mixed in 1:1 ration with LGPIG reporter cells containing sgRNAs targeting the gene of interest. Mixed cell populations were infected with VSV-rWT at MOI 5. The respective percentage of viable (FSC/SSC) mCherry-positive and eGFP-positive cells at the indicated time points was quantified by flow cytometry. Samples were analysed on an LSR Fortessa (BD Biosciences) and data analysis was performed using FlowJo software (Tree Star Inc., USA).

### RNA isolation and qRT-PCR

For qRT-PCR measurement of viral RNA (vRNA), cells were infected with VSV-GFP at MOI 20 and at the indicated time points total RNA was isolated from the samples using RNeasy Kit (Qiagen). RNA was reverse transcribed using random hexamer primers and RevertAid Reverse Transcriptase (Fermentas). qRT-PCR was performed using SensiMix SYBR Green (Bioline) on a QIAGEN Rotor-Gene Q. Results were normalized to the levels of the housekeeping gene *GAPDH* (Suppl. Table 2).

### Viral titer measurement

For the measurement of virus progeny, cells were infected with VSV-GFP at MOI of 2. After incubation with infectious media for 30 min at 37 °C, cells were washed with PBS and media replaced with full IMDM for the remaining duration of the experiment (8 hours in total). Media, containing viral particles, was serially diluted and added to fresh HAP1 wild type cells. Cells were further incubated for 5 hours and the percentage of infected cells was analysed by flow cytometry. Viral titer was calculated using the following formula: U/ml = (cell number * % of GFP^+^ cells)/(volume of virus containing media * dilution factor).

### Antibodies and immunoblotting

The following antibodies were used: V5 (Invitrogen, R960-25,) GAPDH (Santa Cruz, sc-365062), alpha tubulin (Abcam, ab-7291-100), pIRF3 (Cell Signaling, #4947), IRF3 (Cell Signaling, #11904), pSTAT1 (Cell Signaling, #9171), STAT1 (BD Transduction, #610115), p-p65 (Cell Signaling, #3033), p65 (Cell Signaling, #8242), cleaved caspase 3 (Cell Signaling, #9661), cleaved caspase 7 (Cell Signaling, #9491), caspase 8 (Cell Signaling, #9746), caspase 9 (Cell Signaling, #9502), phospho-ERK1/2 T202/Y204 (Cell Signaling, #4370), ERK1/2, (Cell Signaling, #4694), phospho-MEK1/2 S217/221 (Cell Signaling, #9154), MEK1/2 (Cell Signaling, #9126), ARF4 (Proteintech, #11673-1-AP), CHOP (Invitrogen, #MA1-250), GRP78/BiP (BD Biosciences, #610979) and VSV-G (Kerafast, #8G5F11). The following secondary antibodies were used: goat anti-mouse HRP (115-035-003, Jackson ImmunoResearch) and goat anti-rabbit HRP (111-035-003, Jackson ImmunoResearch).

For immunoblotting, whole cell extracts were prepared using RIPA lysis buffer (25 mM Tris/HCl pH 7.6, 150 mM NaCl, 1% NP-40, 1% sodium deoxycholate, 0.1% SDS and one tablet of Roche EDTA-free protease inhibitor per 50 ml) supplemented with Halt phosphatase inhibitor cocktail (Thermo Fisher Scientific #78420) and Benzonase (71205, EMD Millipore). Protein extracts were normalized using the Bradford assay (Bio-Rad). Cell lysates were run on SDS-polyacrylamide gel and transferred to nitrocellulose membranes Protran BA 85 (GE Healthcare). The membranes were incubated with the antibodies indicated above and visualized with horseradish peroxidase-conjugated secondary antibodies using the ECL Western blotting system (Thermo Scientific).

### Immunofluorescence

For immunofluorescence detection of subcellular localization of overexpressed SLC30A1 wild type and mutant fused to mScarlet in HAP1 cells, cells were seeded onto poly-L-lysine hydrobromide (P6282, Sigma-Aldrich)-coated 96-well CellCarrier Ultra plates (PerkinElmer). After an attachment period, cells were stained for the measurement of the intracellular Zn^2+^ levels as described above. Images were acquired on an Opera Phenix automated spinning disk confocal microscope (PerkinElmer).

### Statistical analysis

All quantitative data is presented as the mean ± standard deviation (SD) of one representative experiment performed in technical triplicates. Statistical analysis was performed with GraphPad Prism 7 (GraphPad Software). Details of each statistical analysis are listed in the corresponding figure legends.

## Supplementary information

Supplementary information

Table 1

Table 2

## Data Availability

The datasets generated during and/or analysed during the current study are available from the corresponding author on reasonable request.
