# Supplementary material for: The transporters SLC35A1 and SLC30A1 play opposite roles in cell survival upon VSV virus infection
Source: Sci Rep. 2019 Jul 18;9:10471. doi: 10.1038/s41598-019-46952-9 (PMC6639343; doi:10.1038/s41598-019-46952-9)
Supplement: Supplementary file 1 — Supplementary information [file 41598_2019_46952_MOESM1_ESM.pdf]

SUPPLEMNTARY INFORMATION

The transporters SLC35A1 and SLC30A1 play opposite roles in cell survival upon VSV virus infection

Anna Moskovskich[1], Ulrich Goldmann[1], Felix Kartnig[1], Sabrina Lindinger[1], Justyna Konecka[1], Giuseppe Fiume[1], Enrico Girardi[1*], Giulio Superti-Furga[1,2*]

[1] CeMM Research Center for Molecular Medicine of the Austrian Academy of Sciences, 1090 Vienna, Austria

[2] Center for Physiology and Pharmacology, Medical University of Vienna, 1090 Vienna, Austria

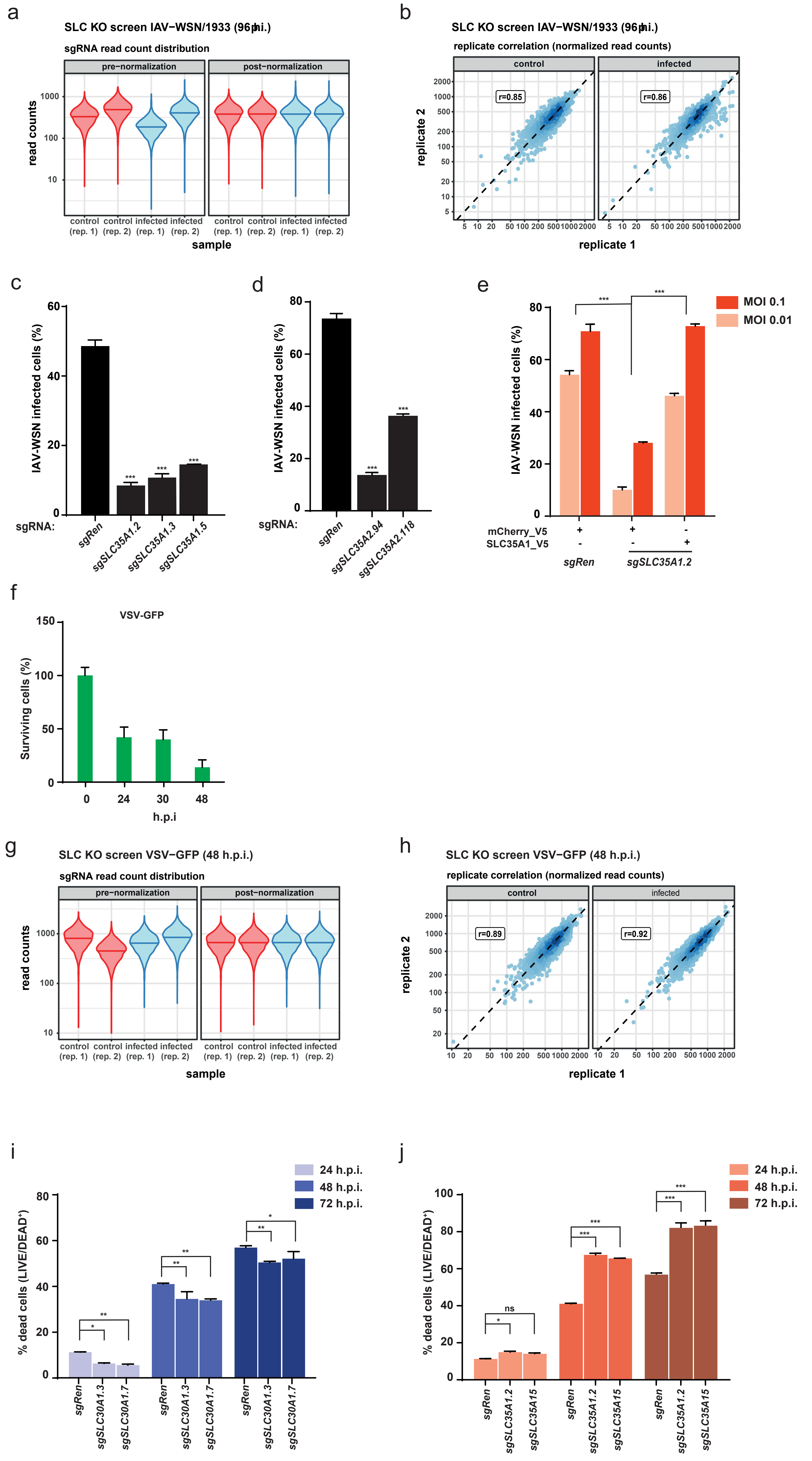
a
SLC KO screen IAV−WSN/1933 (96 h.p.i.)
sgRNA read count distribution
pre−normalization
post−normalization
read counts
control (rep. 1)
control (rep. 2)
infected (rep. 1)
infected (rep. 2)
sample
b
SLC KO screen IAV−WSN/1933 (96 h.p.i.)
replicate correlation (normalized read counts)
control
infected
r=0.85
r=0.86
replicate 2
replicate 1
c
IAV-WSN infected cells (%)
sgRNA:
sgRen
sgSLC35A1.2
sgSLC35A1.3
sgSLC35A1.5
d
IAV-WSN infected cells (%)
sgRNA:
sgRen
sgSLC35A2.94
sgSLC35A2.118
e
MOI 0.1
MOI 0.01
IAV-WSN infected cells (%)
mCherry_V5
SLC35A1_V5
sgRen
sgSLC35A1.2
f
VSV-GFP
Surviving cells (%)
h.p.i
g
SLC KO screen VSV−GFP (48 h.p.i.)
sgRNA read count distribution
pre−normalization
post−normalization
read counts
control (rep. 1)
control (rep. 2)
infected (rep. 1)
infected (rep. 2)
sample
h
SLC KO screen VSV−GFP (48 h.p.i.)
replicate correlation (normalized read counts)
control
infected
r=0.89
r=0.92
replicate 2
replicate 1
i
24 h.p.i.
48 h.p.i.
72 h.p.i.
% dead cells (LIVE/DEAD+)
sgRen
sgSLC30A1.3
sgSLC30A1.7
j
24 h.p.i.
48 h.p.i.
72 h.p.i.
% dead cells (LIVE/DEAD+)
sgRen
sgSLC35A1.2
sgSLC35A15
ns

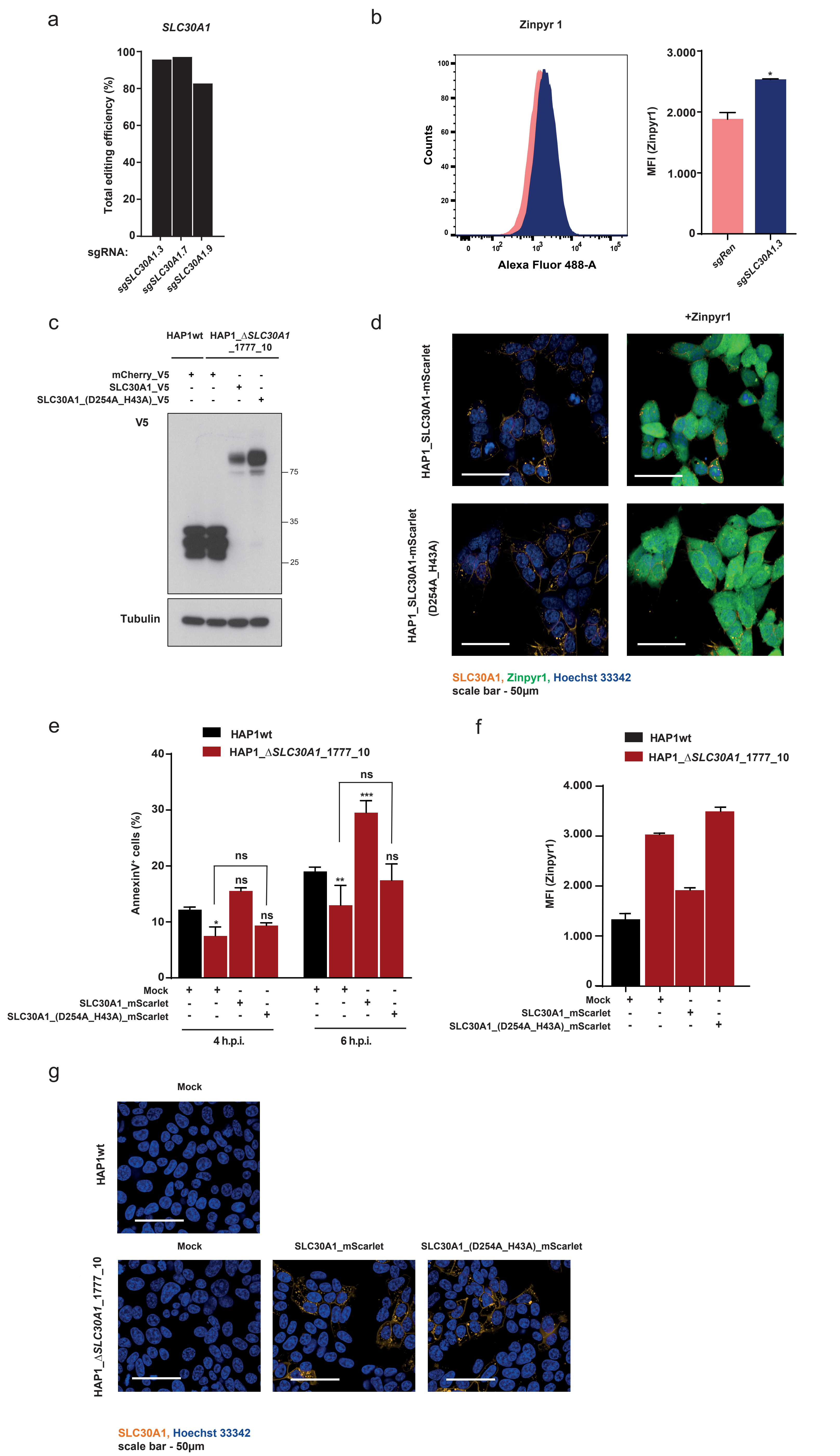
a
SLC30A1
Total editing efficiency (%)
100
80
60
40
20
0
sgRNA:
sgSLC30A1.3
sgSLC30A1.7
sgSLC30A1.9
b
Zinpyr 1
Counts
Alexa Fluor 488-A
MFI (Zinpyr1)
3.000
2.000
1.000
0
*
sgRen
sgSLC30A1.3
c
HAP1wt
HAP1_ΔSLC30A1_1777_10
mCherry_V5
SLC30A1_V5
SLC30A1_(D254A_H43A)_V5
V5
75
35
25
Tubulin
d
+Zinpyr1
HAP1_SLC30A1-mScarlet
HAP1_SLC30A1-mScarlet (D254A_H43A)
SLC30A1, Zinpyr1, Hoechst 33342
scale bar - 50µm
e
HAP1wt
HAP1_ΔSLC30A1_1777_10
AnnexinV+ cells (%)
40
30
20
10
0
ns
***
**
*
Mock
SLC30A1_mScarlet
SLC30A1_(D254A_H43A)_mScarlet
4 h.p.i.
6 h.p.i.
f
HAP1wt
HAP1_ΔSLC30A1_1777_10
MFI (Zinpyr1)
4.000
3.000
2.000
1.000
0
Mock
SLC30A1_mScarlet
SLC30A1_(D254A_H43A)_mScarlet
g
Mock
HAP1wt
HAP1_ΔSLC30A1_1777_10
SLC30A1_mScarlet
SLC30A1_(D254A_H43A)_mScarlet
SLC30A1, Hoechst 33342
scale bar - 50µm

Suppl Fig 3

Moskovskich et al

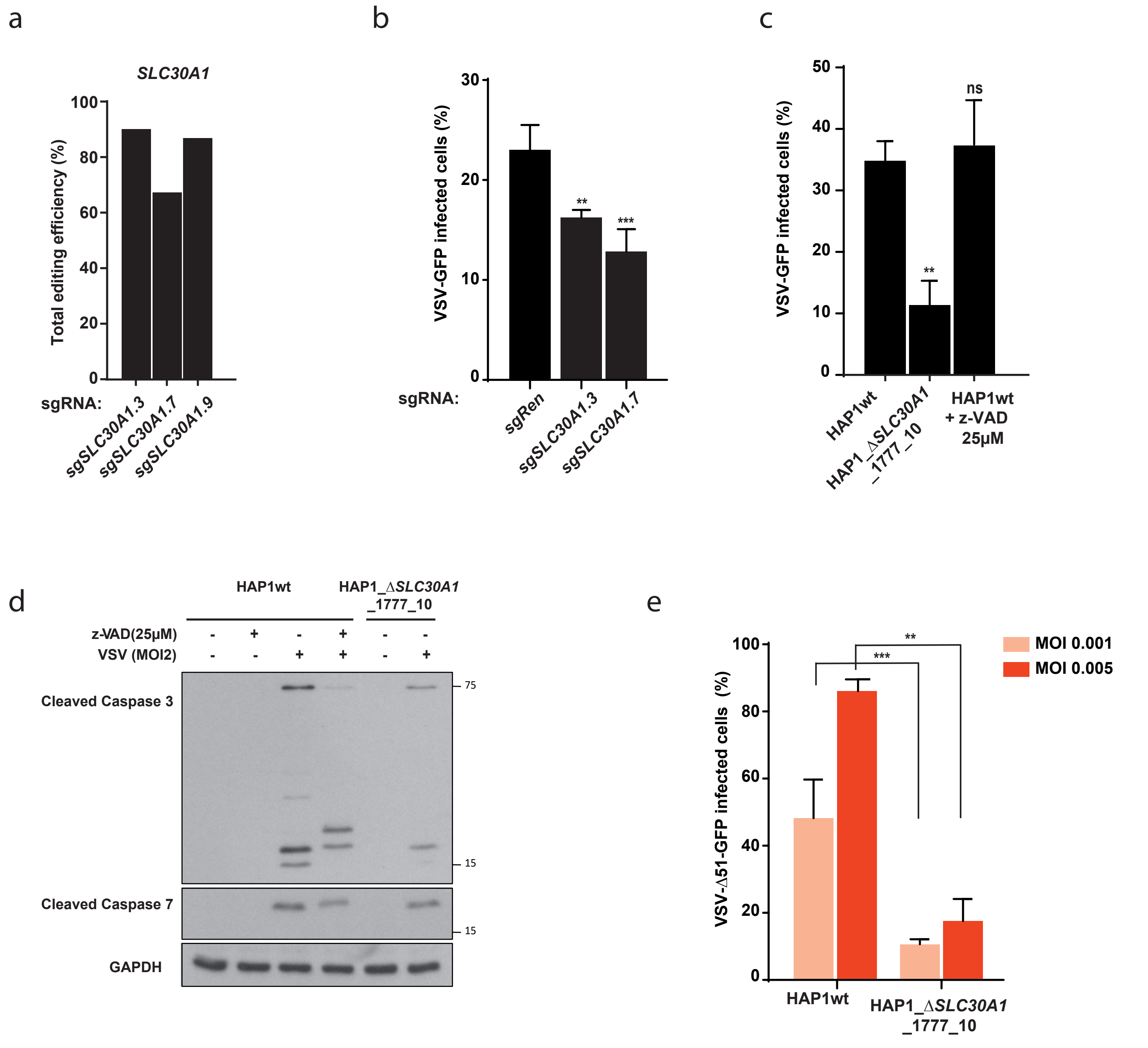

Suppl Fig 4 Moskovskich et al

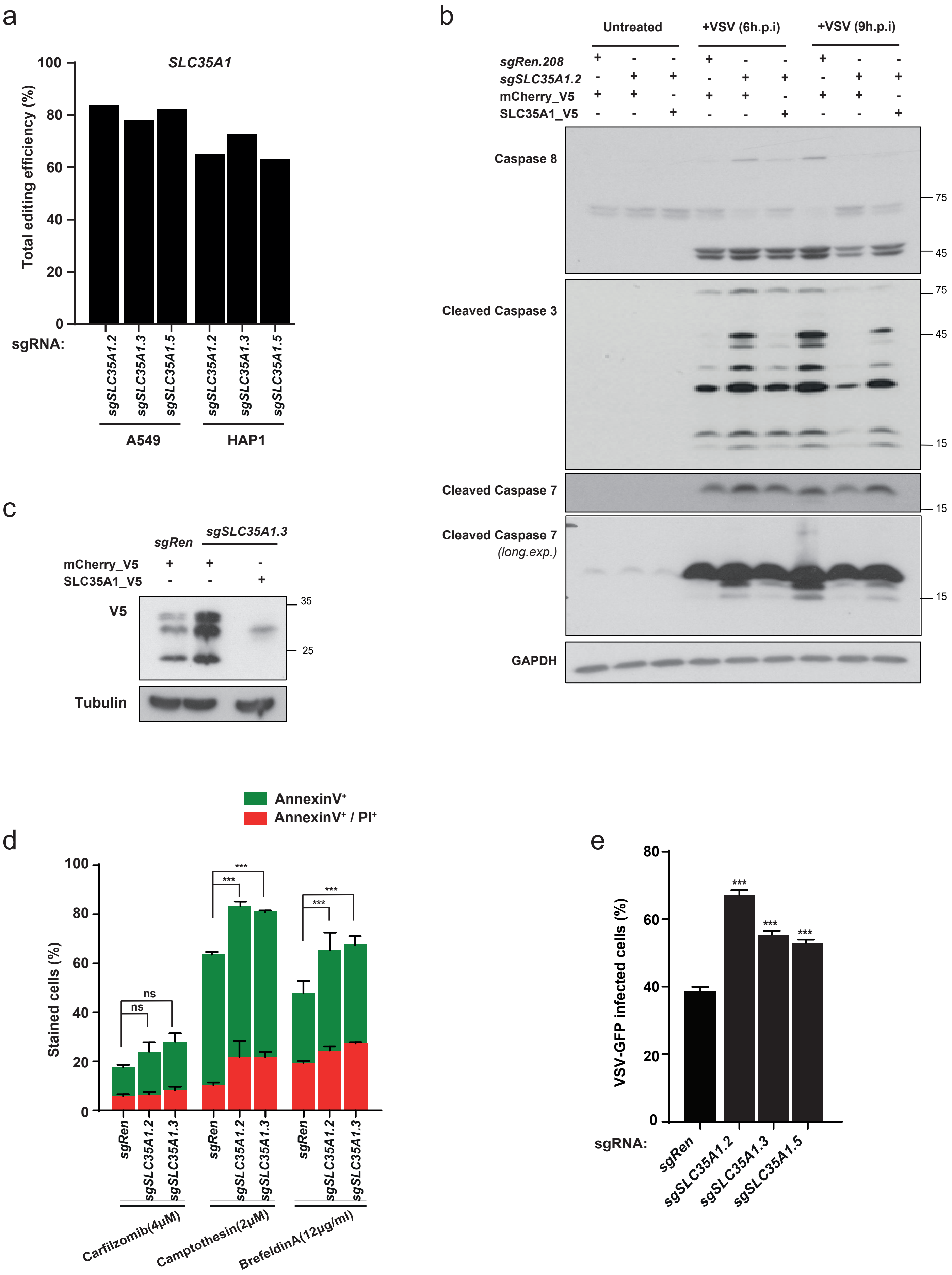

Full lenght immunoblots from Fig 2b

Caspase 9

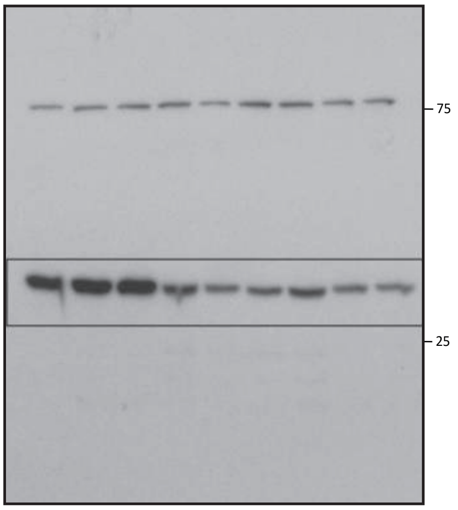

Caspase 9
(long. exp.)

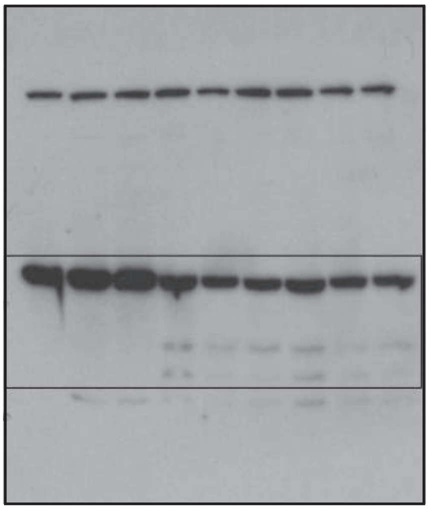

Cleaved Caspase 7

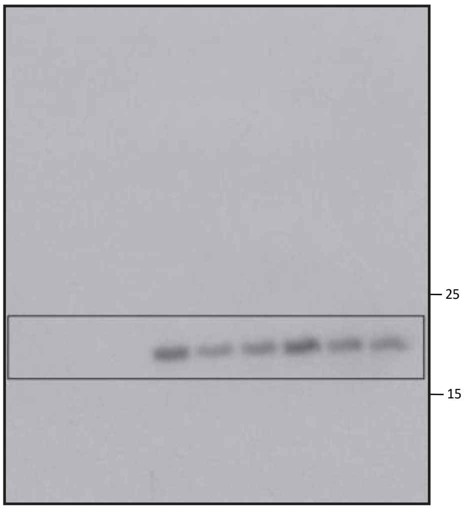

Cleaved Caspase 3

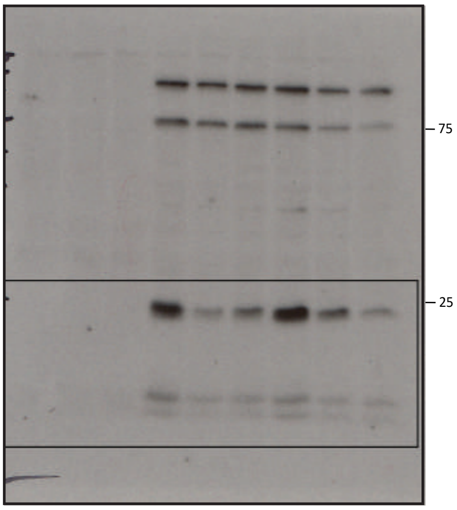

GAPDH

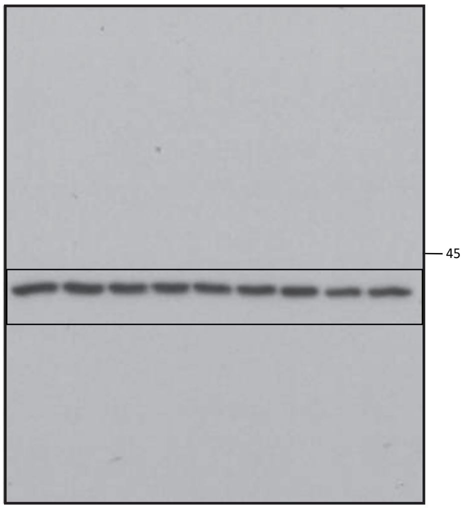

Full lenght immunoblots from Fig 2c

Caspase 8

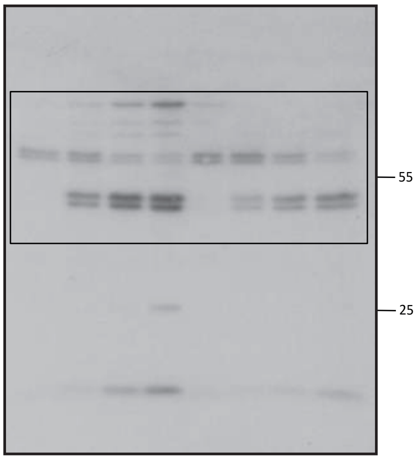

Cleaved Caspase 7

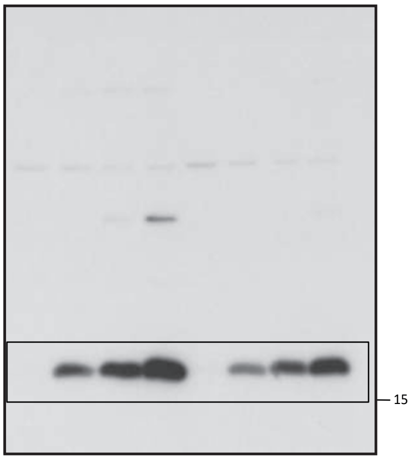

Caspase 9

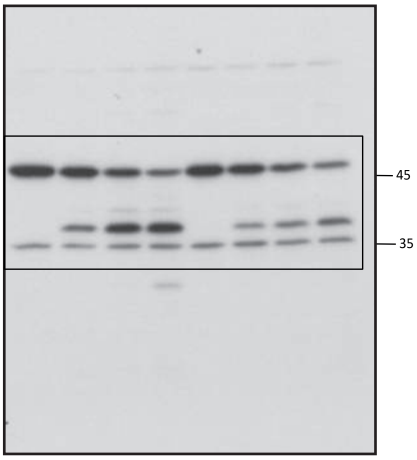

Cleaved Caspase 3

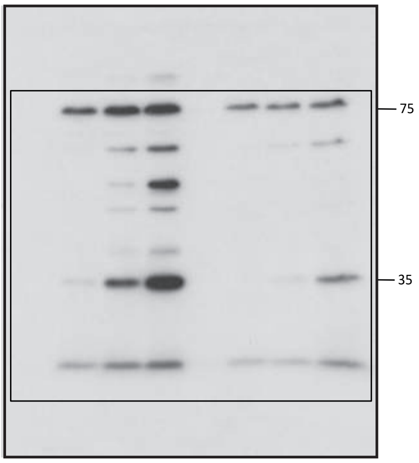

GAPDH

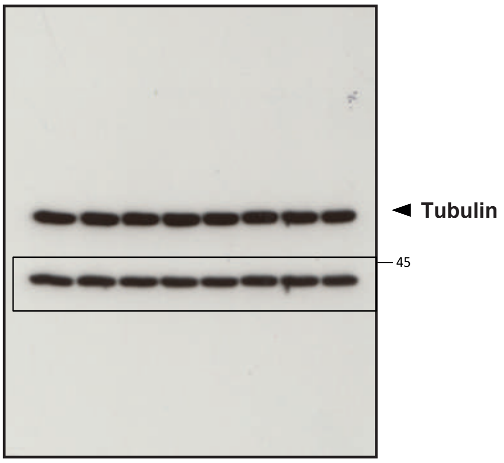

Full lenght immunoblots from Fig 3d

p-IRF3

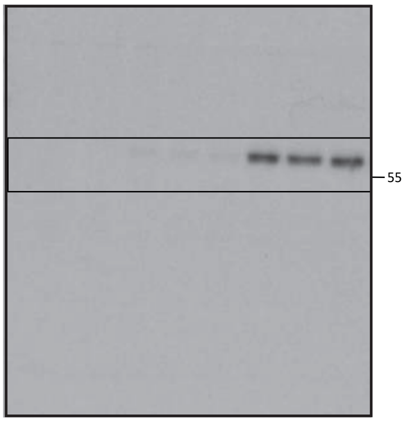

p-IRF3
(long.exp.)

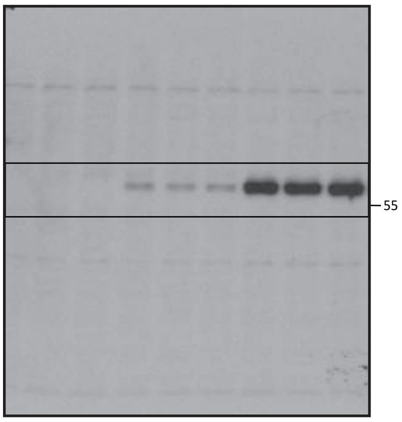

IRF3

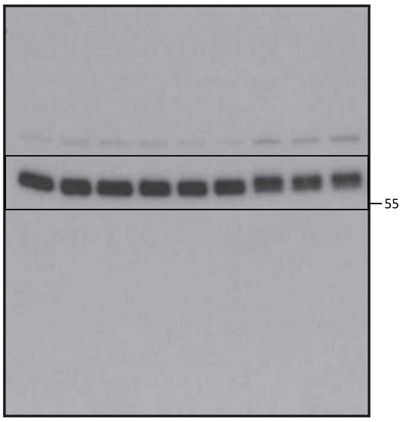

p-STAT1

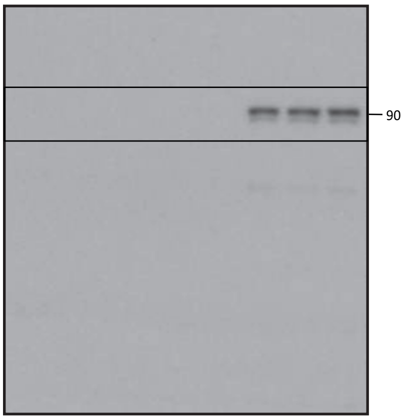

p-STAT1
(long.exp.)

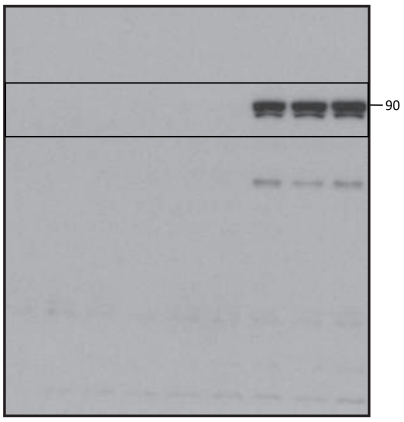

STAT1

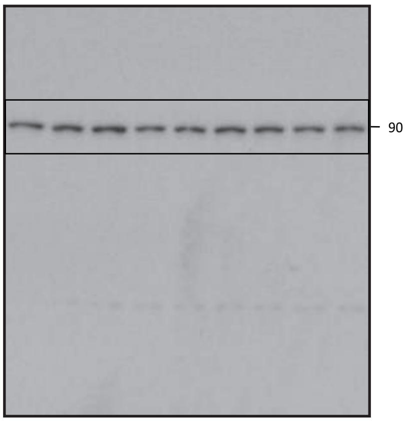

p-p65

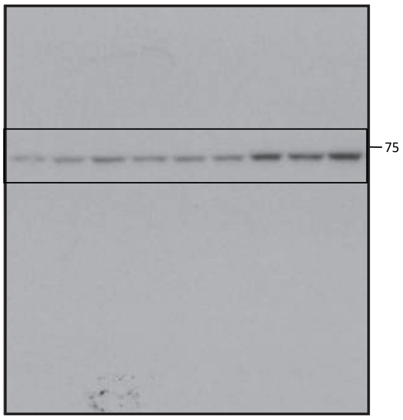

p65

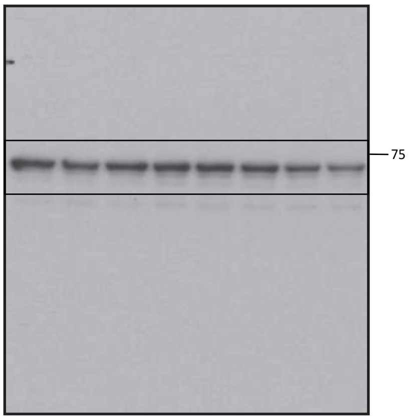

GAPDH

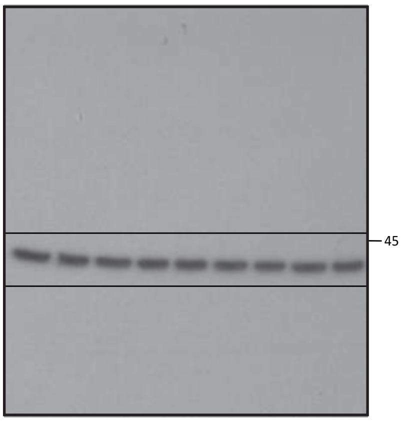

Full lenght immunoblots from Fig 3f

VSV_G

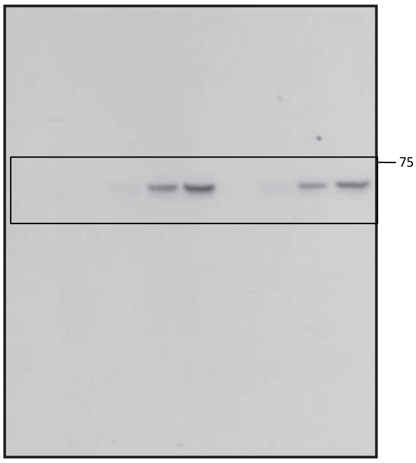

GAPDH

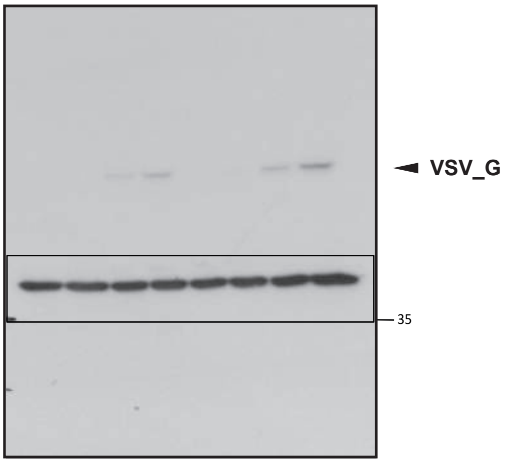

Full lenght immunoblots from Fig 4b

p-MEK

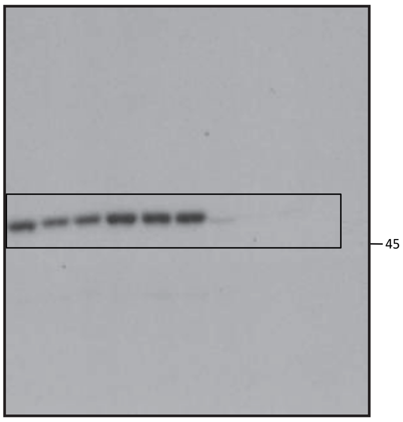

p-MEK
(long.exp)

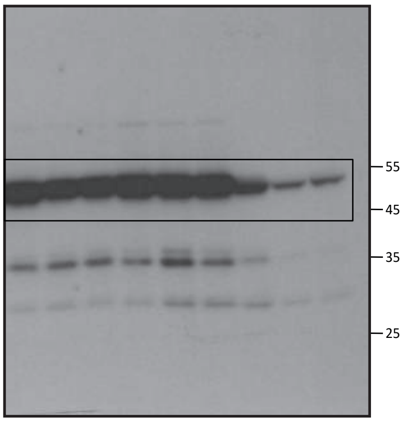

MEK

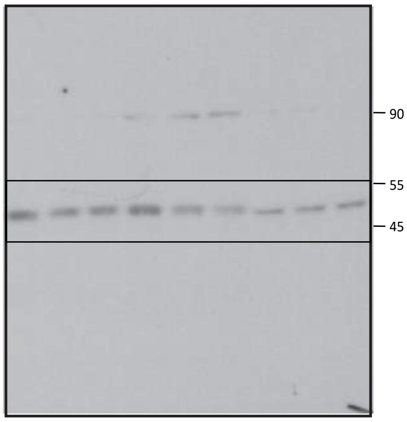

p-ERK

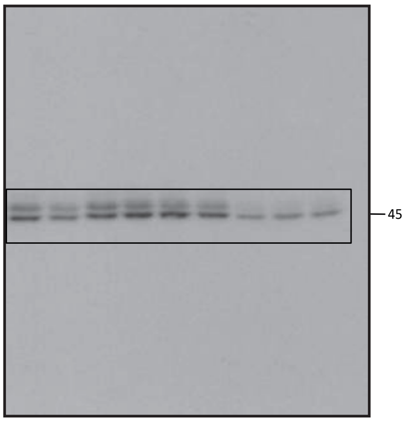

ERK

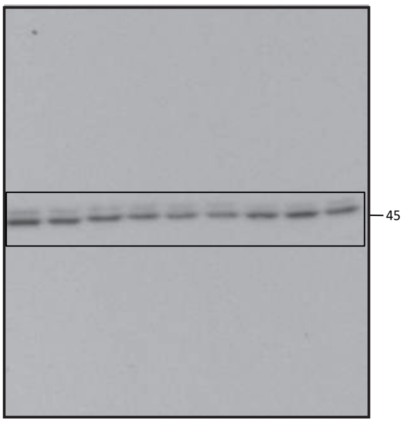

ARF4

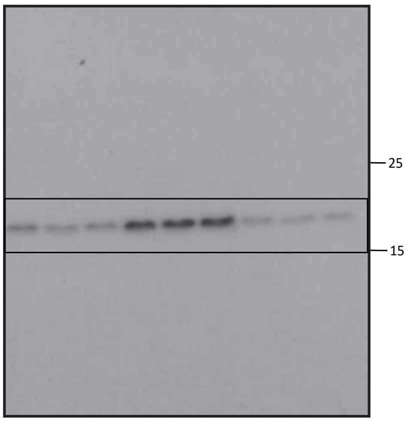

GRP78

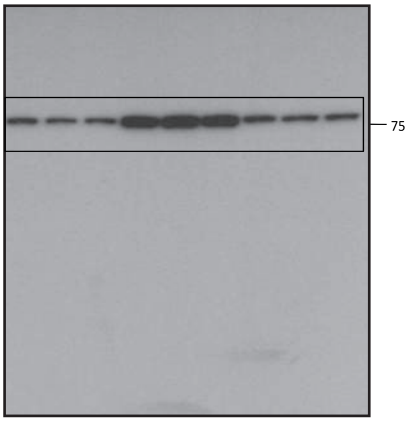

CHOP

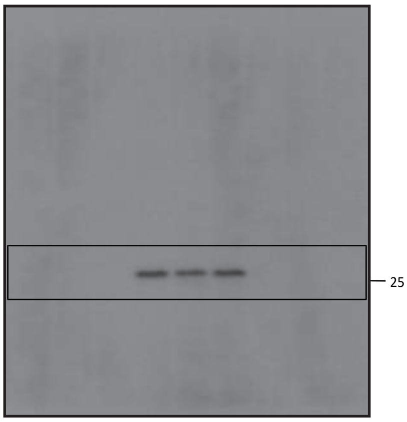

GAPDH

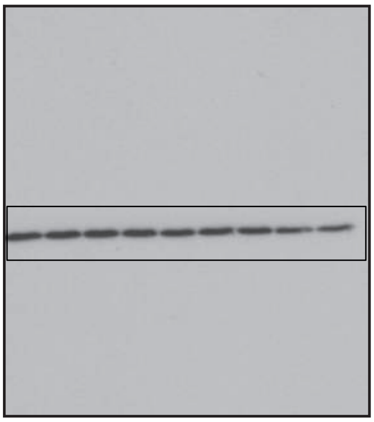

Full lenght immunoblots from Suppl Fig 2c

V5

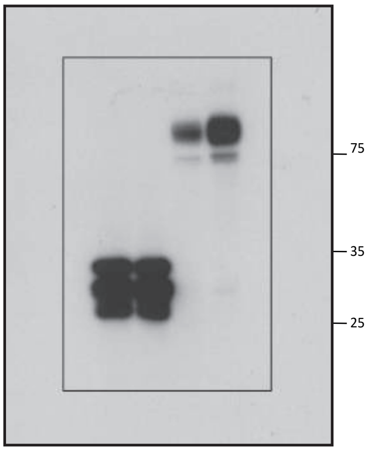

GAPDH

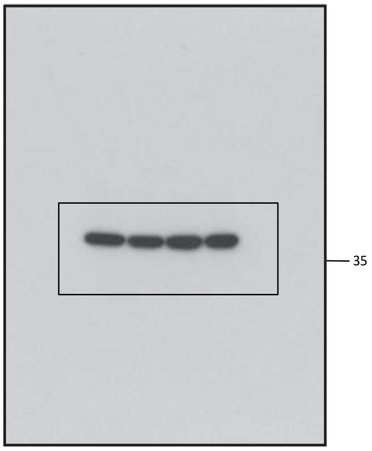

Full lenght immunoblots from Suppl Fig 3d

Cleaved Caspase 3

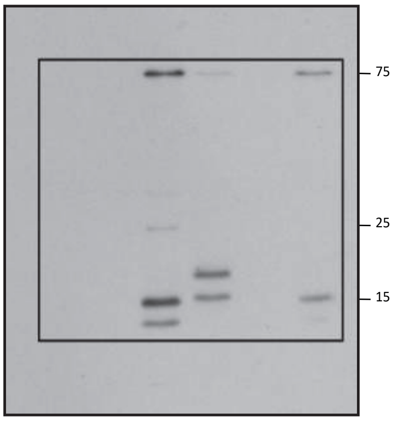

Cleaved Caspase 7

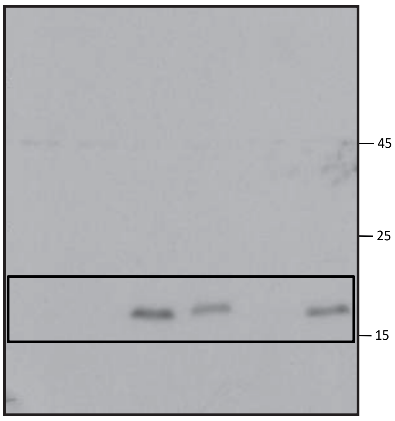

GAPDH

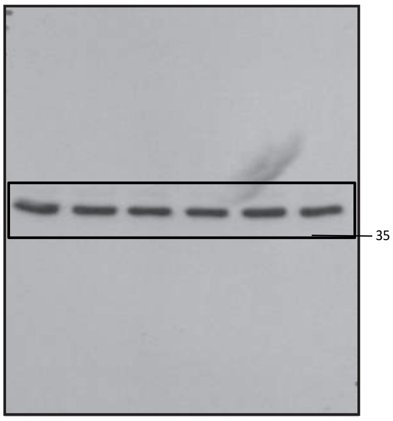

Full lenght immunoblots from Suppl Fig 4b

Caspase 8

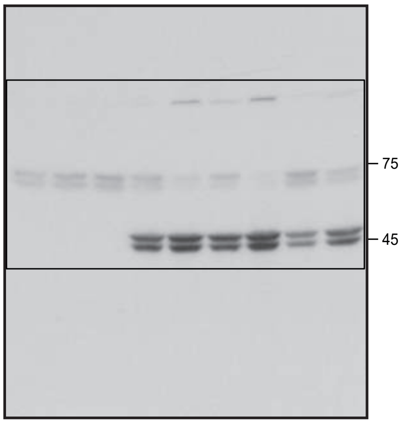

Cleaved Caspase 3

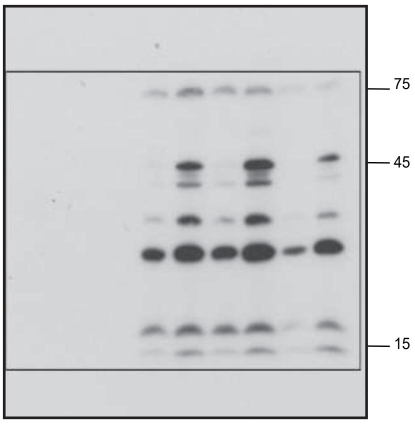

Cleaved Caspase 7

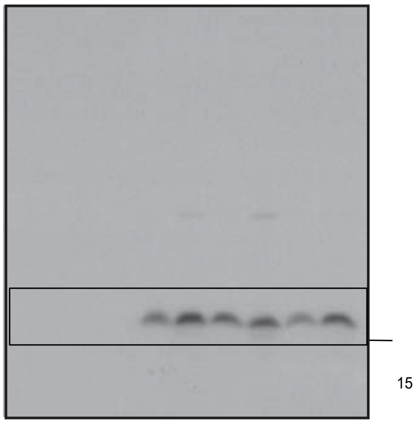

Cleaved Caspase 7
(long.exp.)

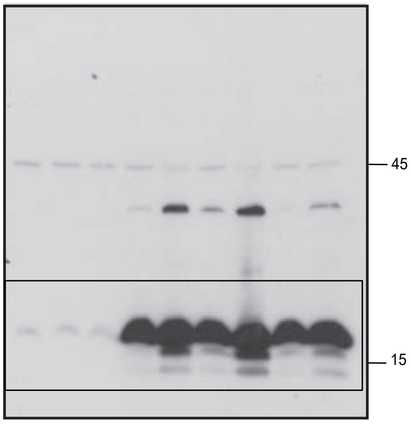

GAPDH

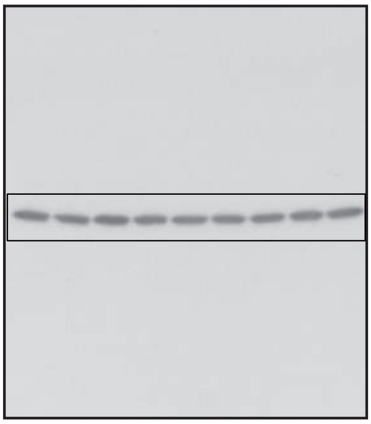

Full lenght immunoblots from Suppl Fig 4c

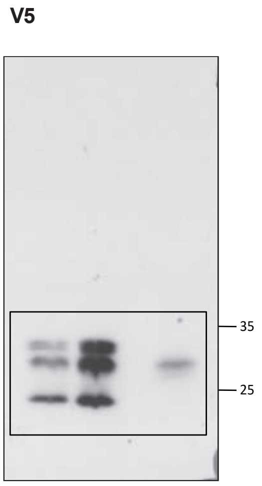

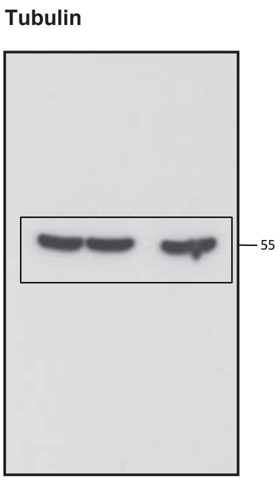

Supplementary Figures

Suppl.Fig. 1 Genetic screens for cell survival upon IAV and VSV infection. a. Violin plots of raw and normalized read counts and **b.** Correlation of normalized read counts across biological replicates in the A549 IAV-WSN/1933 screen. **c-e.** Quantification of flow cytometry-based viral replication assay for HAP1 *SLC35A1* **(c),** *SLC35A2* **(d)** knockout cell lines and cells re-expressing SLC35A1 cDNA **(e)**. Cells were infected with IAV-WSN/1933 at MOI 0.01. At 24 h.p.i. the number of the infected cells was assessed after staining with anti-Influenza Nucleoprotein antibody coupled to AF488. **f.** Assessment of A549 cell death upon infection with wild type VSV-GFP (MOI of 10). Cells were incubated for the indicated time points and relative cell number was determined using crystal violet staining. **g.** Violin plots of raw and normalized read counts and **h.** Correlation of normalized read counts across biological replicates in the A549 VSV-GFP screen. **i** and **j**A549 cells expressing sgRNAs targeting *SLC30A1* (**i**) or *SLC35A1* (**j**) were infected with VSV-GFP at MOI of 5 and at the indicated time points stained with LIVE/DEAD cell viability dye. Positive cells were quantified with flow cytometry. Statistical significance was assessed using one-way ANOVA with Dunnett's (**c** and **d**) or two-way ANOVA with Tukey's (**e**) or Dunnett's (**i** and **j**) tests. Unless otherwise indicated, adjusted P-values in relation to to sgRen control are shown. Data are represented as mean ± SD of one representative experiment out of at least two independent replicates. *: $p \leq 0.05$; **: $p \leq 0.01$; ***: $p \leq 0.001$; ns: not significant.

Suppl.Fig. 2. SLC30A1 loss results in increased intracellular Zn^{2+} concentration and decreased virus-induced cell death. a. Editing efficiency of sgRNAs targeting the *SLC30A1* gene as quantified by the TIDE sequencing method in A549 cells. **b**. Representative FACS histogram and quantification of the A549 SLC30A1 knockout cells stained with Zn^{2+} reactive dye – Zinpyr1. **c.** Representative immunoblot of V5 tagged wild type SLC30A1 and SLC30A1(D254A_H43A) double mutant cDNA expressing cells. Cropped images are shown for conciseness. Full-length blots are presented in Supplementary Figure 5. **d.** Immunofluorescent image of HAP1 wild type cells overexpressing mScarlet-fused wild type SLC30A1 and SLC30A1(D254A_H43A) double mutant stained with Zn^{2+} reactive fluorescent dye Zinpyr1. **e**. Percentage of apoptotic cells in wild type or SLC30A1-deficient HAP1 cells in untrasfected (mock) cells or cells transiently transfected with wild type or transport-deficient SCL30A1 cDNA, upon infection with VSV (MOI 2), as measured with AnnexinV-AF647. Transiently transfected cells (mScarlet^{+}) were compared to the equivalent (mScarlet^{-}, mock) non-transfected cells. **f.** Intracellular Zn^{2+} levels determined using Zinpyr1 fluorescent dye in mock transiently transfected HAP1 cells. Experiment performed once. **g**. Immunofluorescent image of HAP1 wild type cells or HAP1 SLC30A1-deficient clone overexpressing mScarlet-fused wild type SLC30A1 and SLC30A1(D254A_H43A) double mutant. Statistical significance was assessed using two-tailed unpaired Student's t-test (**b**), two-way ANOVA with Sidak's correction **(e)** or one-way ANOVA with Tukey's test (**f**). Unless indicated otherwise P-values in relation to sgRen control (**b**) or

adjusted P-values in relation to mock transfected HAP1wt (**e** and **f**) cells are shown. Data are represented as mean ± SD of one representative experiment out of at least two independent replicates. *: $p \leq 0.05$; **: $p \leq 0.01$; ***: $p \leq 0.001$; ns: not significant.

Suppl.Fig. 3. SLC30A1 in apoptosis and VSV infectivity. **a.** Editing efficiency of sgRNAs targeting the *SLC30A1* gene as quantified by the TIDE sequencing method in HAP1 cells. **b.** Flow cytometry-based VSV-GFP virus replication in *SLC30A1* knockout HEK293T cells (MOI 0.001). **c.** VSV-GFP virus replication in *SLC30A1* knockout HAP1 cells treated with caspase inhibitor z-VAD and infected at MOI of 0.001. The number of GFP^+ cells was quantified with flow cytometry at 14 h.p.i. **d.** Immunoblot analysis of caspase 3 and 7 cleavage in HAP1wt cells stimulated with zVAD and SLC30A1 knockout clone upon infection with VSV-GFP (MOI 2). Cropped images are shown for conciseness. Full-length blots are presented in Supplementary Figure 5. **d.** VSV-Δ51-GFP virus replication in the HAP1 SLC30A1 knockout and wild type cells infected with MOI 0.001 and 0.005 for 14 hours. Statistical significance was assessed using one-way ANOVA with Dunnett's test (**b** and **c**) or two-way ANOVA with Sidak's correction (**e**). Unless otherwise indicated, adjusted P-values in relation to sgRen control or HAP1wt are shown. Data are represented as mean ± SD of one representative experiment out of at least two independent replicates. **: $p \leq 0.01$; ***: $p \leq 0.001$; ns: not significant.

Suppl.Fig. 4. SLC35A1 in apoptosis and VSV infectivity. **a.** Editing efficiency of sgRNAs targeting *SLC35A1* gene as quantified using the TIDE sequencing method in HAP1 and A549 cells. **b.** Pro-apoptotic caspases activation of VSV infected HAP1 *SLC35A1* knockout cells and SLC35A1 cDNA-expressing cells. Cropped images are shown for conciseness. Full-length blots are presented in Supplementary Figure 5. **c.** Representative immunoblot of HAP1 cells expressing V5-tagged wild type SLC35A1. Cropped images are shown for conciseness. Full-length blots are presented in Supplementary Figure 5. **d.** Percentage of apoptotic ($AnnexinV^+$) and necrotic ($AnnexinV^+/PI^+$) cells in the HAP1 cell line expressing sgRNAs against *SLC35A1* and *Renilla luciferase* and stimulation with Brefeldin A (12μg/ml), Carfilzomib (4μM) and Campthotesin (2μM) for 8 hours. **e.** Flow cytometry based VSV-GFP virus replication in *SLC35A1* knockout HEK293T cells (MOI 0.001). Statistical significance was assessed using one-way ANOVA with Tukey's (**d** and **f**) or Dunnett's (**e**) tests. Unless otherwise indicated, adjusted P-values in relation to sgRen control are shown. Data are represented as mean ± SD of one representative experiment out of at least two independent replicates. **: $p \leq 0.01$; ***: $p \leq 0.001$; ns: not significant.

Suppl.Fig. 5. Full blots used in this study.

Tables

- **Suppl Table1**: Guide RNA read count tables from SLC knoxkout screens
- **Suppl Table2**: Primers (qPCR, sequencing) and sgRNAs used in the study
